# Assessment of Dried Serum Spots (DSS) and Volumetric-Absorptive Microsampling (VAMS) Techniques in Therapeutic Drug Monitoring of (Val)Ganciclovir—Comparative Study in Analytical and Clinical Practice

**DOI:** 10.3390/ijms25168760

**Published:** 2024-08-12

**Authors:** Arkadiusz Kocur, Agnieszka Czajkowska, Mateusz Moczulski, Bartłomiej Kot, Jacek Rubik, Tomasz Pawiński

**Affiliations:** 1 Department of Drug Chemistry, Pharmaceutical and Biomedical Analysis, Medical University of Warsaw, Banacha 1, 02-097 Warsaw, Poland; tomasz.pawinski@wum.edu.pl; 2 Therapeutic Drug Monitoring, Clinical Pharmacokinetics and Toxicology Laboratory, Department of Clinical Biochemistry, The Children’s Memorial Health Institute in Warsaw, Dzieci Polskich 20, 04-730 Warsaw, Poland; 3 Student Scientific Association “Drug” in Department of Drug Chemistry, Pharmaceutical and Biomedical Analysis, Medical University of Warsaw, Banacha 1, 02-097 Warsaw, Poland; 4 Department of Nephrology, Kidney Transplantation and Hypertension, The Children’s Memorial Health Institute, Dzieci Polskich 20, 04-730 Warsaw, Poland; j.rubik@ipczd.pl

**Keywords:** ganciclovir, valganciclovir, cytomegalovirus, dried serum spots, VAMS, LC-MS/MS

## Abstract

Ganciclovir (GCV) and its prodrug valganciclovir (VGCV) are antiviral medications primarily used to treat infections caused by cytomegalovirus (CMV), particularly in immunocompromised individuals such as solid organ transplant (SOT) recipients. Therapy with GCV is associated with significant side effects, including bone marrow suppression. Therefore, therapeutic drug monitoring (TDM) is mandatory for an appropriate balance between subtherapeutic and toxic drug levels. This study aimed to develop and validate three novel methods based on liquid chromatography-tandem mass spectrometry (LC-MS/MS) for GCV determination in serum (reference methodology), dried serum spots (DSS), and VAMS-Mitra™ devices. The methods were optimized and validated in the 0.1–25 mg/L calibration range. The obtained results fulfilled the EMA acceptance criteria for bioanalytical method validation. Assessment of DSS and VAMS techniques extended GCV stability to serum for up to a minimum of 49 days (at room temperature, with desiccant). Developed methods were effectively evaluated using 80 clinical serum samples from pediatric renal transplant recipients. Obtained samples were used for DSS, and dried serum VAMS samples were manually generated in the laboratory. The results of GCV determination using serum-, DSS- and VAMS-LC-MS/MS methods were compared using regression analysis and bias evaluation. The conducted statistical analysis confirmed the interchangeability between developed assays. The DSS and VAMS samples are more accessible and stable during storage, transport and shipment than classic serum samples.

## 1. Introduction

**Figure 1:**
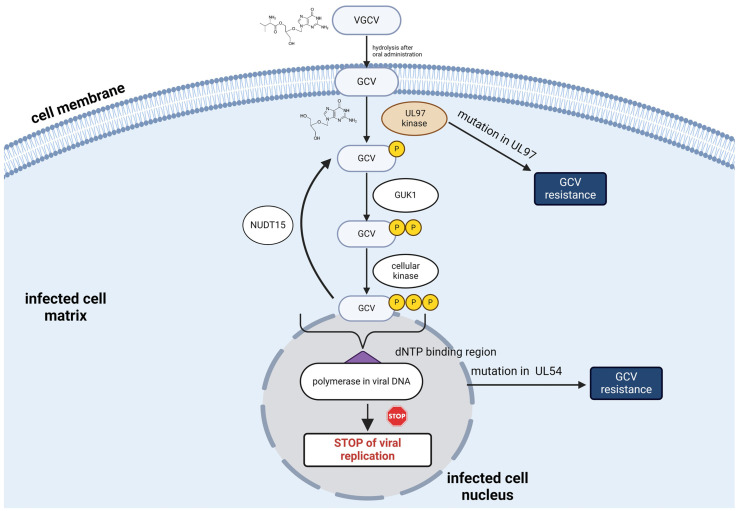
Molecular mechanisms of (val)ganciclovir action in CMV-infected cells. Mutations in UL97 and UL54 are responsible for GCV resistance. GCV—ganciclovir, VGCV—valganciclovir, GUK1—guanylate kinase 1, NUDT15—nudix hydrolase 15, dNTP—deoxynucleotide triphosphate. Created using bioRender.com (accessed on 8 August 2024).

Cytomegalovirus (CMV) infection (CMV disease) in solid organ transplant (SOT) recipients is one of the leading reasons for chronic and acute graft rejection [1]. Additionally, CMV pneumonia may cause up to 50% mortality in this group of patients. Therefore, appropriate viral detection (molecular techniques) and optimal antiviral pharmacotherapy are necessary for virus eradication [1,2,3]. Ganciclovir (9-(1,3-dihydroxy-2-propoxymethyl)guanine) and its prodrug, valganciclovir (VGCV; L-valyl ester of GCV), are the gold standards for the prevention and treatment of CMV infection in case of SOT recipients. Additionally, both agents are included in the WHO Model List of Essential Medicines [3]. After oral administration, VGCV is rapidly hydrolysed to its active form, GCV. The molecular mechanism of action of GCV is shown in Figure 1. Notably, mutations in UL97 and UL54 genes caused resistance to GCV treatment, which may lead to the need for additional drug dosing adjustments [3,4,5,6,7].

Therapeutic drug monitoring (TDM) of GCV is still discussed. It is helpful, especially for dosing adjustments based on potential toxic adverse effects. These include myelosuppression and thrombocytopenia (in cases of drug overexposure). On the other hand, sub-therapeutic GCV levels may lead to antiviral therapy failure. Monitoring of GCV concentration is beneficial for dose adjustment in the pediatric population [7]. Additionally, the GCV and VGCV doses routinely used in the pediatric population after renal transplantation depend on various individual factors, i.e., BSA (body surface area), creatinine clearance, and the reason for pharmacotherapy introduction (prophylaxis, pre-emptive strategies, or active CMV infection) [2,3,4,5,6,7].

Owing to potential toxic side effects (bone marrow suppression) associated with the use of (val)ganciclovir, as well as viral mechanisms of resistance to this drug, monitoring of drug levels is recommended, especially in children, with recommended therapeutic concentrations of 1–3 mg/L for C_0_ (trough concentration) and 5–12 mg/L for C_max_ (maximum (peak) concentration) [3,4,5,6,7]. Ideally, the appropriate pharmacokinetics parameter characterised by drug exposure is AUC_0–24_ (area under the curve time—concentration), for which the target value should be within 40–60 mg∙h/L. If C_0_ is greater than 1 mg/L, a daily dose or dosing interval modification may be necessary. Routinely, peak concentration adjustment is not usually required with optimal C_0_ and an appropriate antiviral response controlled by CMV viremia or viruria level [5,6,7,8].

Chromatography is the method of choice for GCV quantification in blood samples during clinical TDM. The estimated plasma concentration of GCV is relatively high; therefore, high-performance liquid chromatography with ultraviolet detection (HPLC/UV) seems to be sufficient. However, liquid chromatography-tandem mass spectrometry (LC-MS/MS) is more precise and practical, mainly when the trough concentration is relatively low. The high sensitivity of the LC-MS/MS technique make it a valuable tool for pharmacokinetic profile sample determination, especially when VGCV is not fully hydrolysed and, as a consequence, still present in plasma [5].

Internal standardisation is the most frequently used approach for method calibration. The EMA guidelines for bioanalytical method validation propose using stable isotope labelled internal standard as a reference in LC-MS/MS method validation. On the other hand, some analogue-related internal standards (ARIS) or unrelated internal standards (URIS) may be attractive approaches for bioanalytical method validation. This is primarily due to the lower unit price of solid standards and sometimes higher chemical purity and ubiquitous availability.

The most useful matrix for GCV determination is plasma or serum because this drug binds to proteins at 1–2% [1]. Owing to limited TDM laboratories where GCV may be determined, ensuring high stability of the analyte in the matrix may be required. Appropriate sample storage is a crucial point of the preanalytical process; therefore, microsampling techniques such as dried serum spot (DSS), dried plasma spot (DPS), and volumetric-absorptive microsampling (VAMS) help improve sample stability. The stability of analytes are often higher in dried matrix than in liquid blood or their fractions. It seems that the drying process may stop enzymatic processes, degradation, and hydrolysis. Additionally, the limited amount of water in the sample may reduce the risk of adverse processes of sample degradation caused by the microbiome [9]. The techniques mentioned are subject to the hematocrit effect (HE), which is characteristic of DBS (dried blood spot). The weakness of those approaches is that whole blood must be collected via classic venipuncture, and subsequently, the obtained serum is manually transferred for microsample generation.

The present study aimed to develop and validate a novel LC-MS/MS method for determining GCV in serum, DSS, and dried serum VAMS samples (s-VAMS). Extended stability testing was performed, and validated methods were assessed in clinical practice. Additionally, during reference LC-MS/MS method validation for GCV determination in human serum, two different approaches were used for assay calibration, namely based on SIL-IS (GCV-d_5_) and ARIS (aciclovir, ACV).

To the best of our knowledge, the present study is the first application of the Whatman 903 Proteinsaver Cards and VAMS device for storing serum samples for GCV determination.

## 2. Results

### 2.1. Method Development and Optimisation

**Figure 2:**
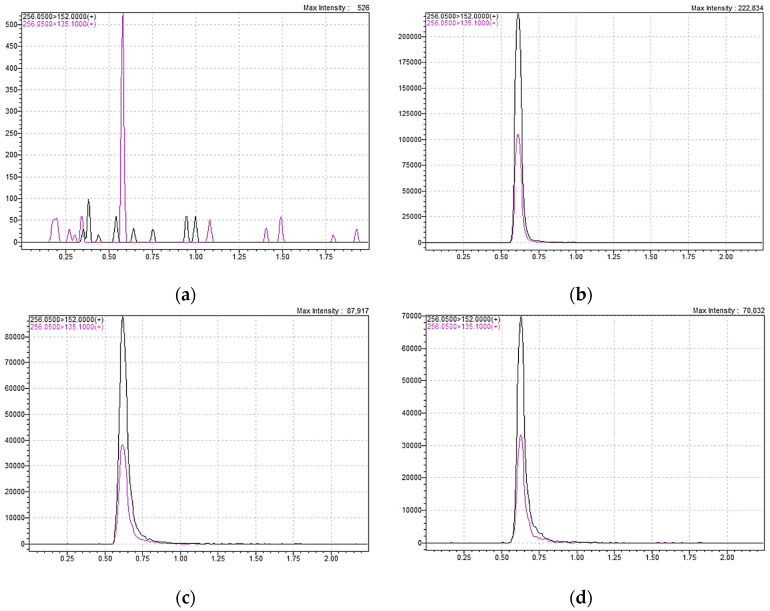
Representative chromatograms of GCV (retention time = 0.63 min): (**a**) blank sample; (**b**) serum patient sample—8.66 mg/L GCV concentration; (**c**) dried serum spot patient sample—8.81 mg/L GCV concentration; (**d**) s-VAMS patient sample—8.33 mg/L GCV concentration.

The present study optimised and validated three LC-MS/MS methods for GCV determination in serum, DSS and VAMS. Chromatographic and mass spectrometry conditions were optimised experimentally (more information in Section 4.3). The total gradient flow rate has been set experimentally as 0.7 mL/min, with water/acetonitrile with formic acid addition (0.1%) assessment. Total LC and MS program run time was set to 2 min. The analyte (GCV) and SIL-IS (GCV-d_5_) were observed on chromatograms at 0.63 min, while ARIS (ACV) was detected at 0.72 min. The optimal temperature for chromatographic column maintenance was set to 30°C given that compound peaks split at higher temperatures. Based on appropriate assay sensitivity, the sample injection volume was set to 0.1 µL for serum samples and 1 µL for DSS and VAMS samples. Representative chromatograms of GCV determination in used matrices are presented in Figure 2.

To optimize analyte extraction from DSS and VAMS samples, different solvents were tested—water, methanol, methanol/water mixture (50:50, *v*/*v*), acetonitrile, acetonitrile: water mixture (50:50, *v*/*v*). Ultimately, a methanol/water mixture combined with an ultrasonication and vortexing sequence was used for analyte extraction from DSS and VAMS samples. Protein precipitation (PPT) methods were tested under various conditions and solvents for optimal sample purification. The optimal solvent for PPT for the method used in the study was a mixture of 0.1 M ZnSO_4_ with acetonitrile (50:50, *v*/*v*).

### 2.2. Analytical Validation

For method validation, EMA (European Medicines Agency) and FDA (Food and Drug Administration) guidelines for bioanalytical method validation were assessed [10,11,12,13]. For matrix effect (ME) evaluation, the approaches proposed by Taylor et al. and Matuszewski et al. were used [14,15].

During serum-LC-MS/MS method validation, GCV-d_5_ and ACV were used for calibration comparison. ACV yielded better validation results and was used to validate alternative sampling strategies (DSS and s-VAMS) LC-MS/MS methods. Calibration models (weighted 1/x) and mean R^2^ coefficients (arithmetic mean ± SD) were determined based on six calibration curve analyses for each method (including IS comparison for serum-LC-MS/MS method validation). The method calibration equations generated were as follows:

(1) serum-LC-MS/MS method:
  (a) IS: GCV-d_5_ − y = 0.032x + 0.004 (R^2^ = 0.993 ± 0.005),
  (b) IS: ACV − y = 0.024x + 0.001 (R^2^ = 0.995 ± 0.003).
(2) DSS-LC-MS/MS method (IS_ACV_):

y = 0.036x + 0.002 (R^2^ = 0.993 ± 0.004),

(3) s-VAMS-LC-MS/MS method (IS_ACV_):

y = 0.038x + 0.001 (R^2^ = 0.997 ± 0.002),

where y is GCV/IS peaks area ratio, and x is GCV nominal concentration.

No interferences were observed during analyses—the chromatographic signals of interfered or endogenous compounds were lower than 15% of the analyte peak at LLOQ (lower limit of quantification). LOD (limit of detection) and LLOQ were determined experimentally based on signal-to-noise ratio (S/N) calculation to be 0.008 mg/L and 0.05 mg/L, respectively, for all validated methods.

The accuracy and precision parameters were evaluated at four calibration levels using quality controls (QCs), as follows: LLOQ (0.10 mg/L), MQC_1_ (0.75 mg/L), MQC_2_ (7.50 mg/L), and HQC (20.00 mg/L). Both parameters were tested in within-run (intra-day) and between-day (inter-day) experiments and subsequently calculated based on ten repetitions for the above QCs for each method. Satisfactory results were obtained for accuracy (acceptance criteria: 85–115% of nominal value) and precision (CV_%_ < 15%, except LLOQ < 20%) validation parameters evaluation in the case of each LC-MS/MS method (Table 1).

Satisfactory GCV stability in samples stored at 4 °C in an autosampler was obtained (Table 2). This validation parameter was tested on the initial day (T = 0) and after 24, 72, and 120 h. Only in the case of the LLOQ level of GCV determination in DSS samples was the stability subnormal (the acceptance criterion is accuracy within 85–115% of nominal value) after 72 and 120 h of observation.

For each validated method, a bench-top (stop-work) experiment was performed at two QC levels: MQC_1_ and MQC_2_ in standard procedure and with a 2-h stop before and after extraction (−2 h and +2 h in Table 3, respectively). The mean stability of the analytes, expressed as mean values with standard deviation (SD) and percentage accuracy, was acceptable according to EMA validation criteria [11]. All experiments were performed six times for each examined matrix using LC-MS/MS methodology.

Following published guidelines and matrix effect (ME) evaluation protocols, the post-extraction experiment was performed in sextuplicate using four levels of QCs spiked into each evaluated matrix [11,12,13,14,15]. Additionally, the process efficiency (PE) and absolute recovery (AR) parameters were calculated using formulas proposed by Taylor et al. [14] and Matuszewski et al. [15]. However, in some cases, the ME was higher than ± 15% (EMA acceptance criteria not fulfilled), but for the GCV/IS peak area ratio, the above-mentioned acceptance criteria were fulfilled. Additionally, no significant ME (including intra-individual ME) was observed during the analysis. The summarised results of ME, PE, and AR evaluation are presented in Table 4.

The crucial point of microsampling techniques based on dried serum is long-term stability examination after the matrix sample is manually put into Whatman paper or absorbed by VAMS tips. A long-term stability experiment for GCV (using ACV as IS) was performed for DSS and s-VAMS techniques at two QC levels in sextuplicate. Stability was evaluated by systematically assaying samples (stored at RT) after 7, 14, 28, 35 and 49 days of initial analysis (T = 0). The stability was satisfactory for both microsampling techniques after 7 weeks of observation, but slightly better results were observed in the case of dried serum VAMS samples (Table 5).

Additionally, the stability of GCV and ISs (GCV-d_5_ and ACV) in MQC_1_ and MQC_2_ QC working solutions was satisfactory during long-term storage at −20 °C (12 weeks). The percentage stability values at tend of the observation period were 99.25%, 95.02%, and 98.26% for GCV, GCV-d_5_, and ACV, respectively.

### 2.3. Patient Sample Determination

The serum samples were obtained from pediatric renal transplant recipients via classic venipuncture whole blood collection in 1.2-mL test tubes with a clot activator (Sarstedt, Nümbrecht, Germany). Part of the sample (100 µL) was analysed using the HPLC/UV method (high-performance liquid chromatography with ultraviolet detection) as routine GCV determination under routine CMV-antiviral therapy monitoring at the Children’s Memorial Health Institute in Warsaw, Poland. Eighty samples were obtained from 40 patients before and two hours after oral VGCV administration (concentration C_0_, trough level and C_max_, peak level, respectively). The residue of the serum sample (100 µL) was used for GCV determination using serum-, DSS-, and s-VAMS-LC-MS/MS methods. DSS and s-VAMS samples were prepared in the TDM laboratory manually based on a specified serum amount or quantitative absorption (35 µL for DSS and 10 µL for the s-VAMS method). The mean GCV concentrations for each method (with minimum and maximum values) were calculated as follows:

(1) serum-LC-MS/MS (GCV-d_5_ as IS)—4.164 (0.054–19.854) mg/L,
(2) serum-LC-MS/MS (ACV as IS)—4.185 (0.039–19.104) mg/L,
(3) DSS-LC-MS/MS (ACV as IS)—4.167 (0.014–19.960) mg/L,
(4) s-VAMS-LC-MS/MS (ACV as IS)—4.402 (0.039–19.760) mg/L,
(5) HPLC/UV (ACV as IS)—4.003 (0.100–21.180) mg/L.

It should be noted that in case of HPLC/UV method, 7.50% of the obtained results were below LOD, whereas in the method using LC-MS/MS, all measured results were higher than LOD and LLOQ.

Following the defined target ranges for C_0_ (1–3 mg/L) and C_max_ (5–12 mg/L) and based on obtained results using validated methods, the number of samples falling in the above ranges were calculated as follows:

(1) serum-LC-MS/MS (GCV-d_5_ as IS)—40% in the C_0_ range; 57.5% in the C_max_ range,
(2) serum-LC-MS/MS (ACV as IS)—42.5% in the C_0_ range, 65% in the C_max_ range,
(3) DSS-LC-MS/MS (ACV as IS)—42.5% in the C_0_ range, 70% in the C_max_ range,
(4) s-VAMS-LC-MS/MS (ACV as IS)—45% in the C_0_ range, 75% in the C_max_ range,
(5) HPLC/UV (ACV as IS)—45% in the C_0_ range, 77.5% in the C_max_ range.

**Figure 3:**
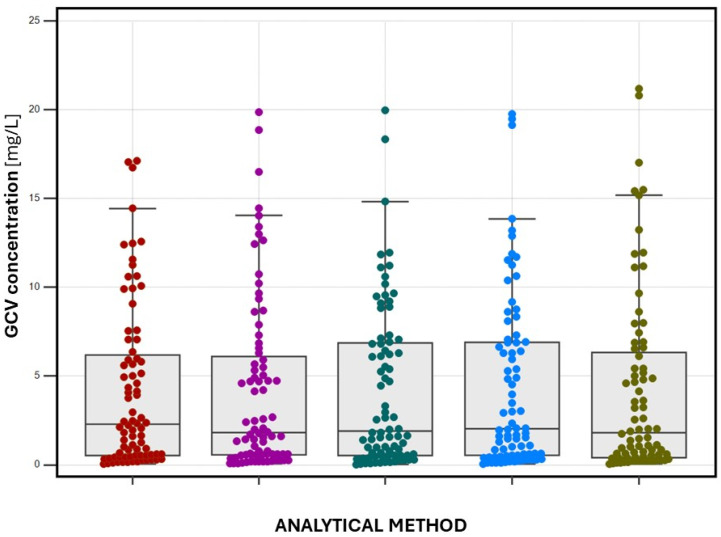
Results of GCV determination using chromatographic methods expressed as bar/dotted graph with whiskers. Methods are marked as follows: red dots (serum-LC-MS/MS with GCV-d_5_ as internal standard), pink dots (serum-LC-MS/MS with ACV as internal standard), green dots (dried serum spots, DSS-LC-MS/MS with ACV as internal standard), blue dots (dried serum in VAMS-LC-MS/MS with ACV as internal standard), green dots (serum-HPLC/UV with AVC as internal standard). GCV—ganciclovir.

The summarised assay results are presented as a graph in Figure 3.

### 2.4. Cross-Validation and Methods Comparison

The results obtained after clinical sample determination using LC-MS/MS and HPLC/UV methods underwent statistical evaluation according to cross-validation and methods comparison. The proper tools for analytical method comparison are regression analysis (Passing-Bablok or Deming model) and bias evaluation using the Bland-Altman methodology. Additionally, correlation methodologies such as Pearson’s and Spearman rank correlation coefficients are sometimes used. The present study’s GCV concentration paired results were statistically analysed (Table 6). Methods are considered comparable when zero (0) is within the confidence interval (95% CI) for intercept and one (1) is within the CI for slope. Passing-Bablok regression may be used for a minimum of 40 paired samples. The systematic bias between two methods is expressed as a percent, with 95% CI. Bias between methods is negligible when this percent is lower than 20% for a minimum of 67% of paired samples [16,17].

## 3. Discussion

This study successfully validated and clinically applied novel, fast, and sensitive LC-MS/MS methods for GCV determination in serum (reference method), DSS, and VAMS. The reference method was also compared with the routine HPLC/UV assay used for TDM. Validated methods were statistically compared using statistical tools: Bland-Altman bias evaluation and Passing-Bablok regression calculation. We proposed a simple extraction process with protein precipitation concurrent with published literature [18,19,20,21,22,23,24,25]. This approach is more applicable to routine practice than complex extraction methodologies such as LLE and SPE.

The literature includes a few developments in chromatographic methods and their application to clinical practice [18,19,20,21,22,23,24]. LC-MS/MS is considered the gold standard for TDM of various analytes, including drugs [25,26,27] Therefore, we decided to validate a reference method for GCV determination in a small amount (35 µL) of serum. A developed and validated reference method was used as a point of comparison for alternative sampling strategies (DSS and VAMS). However, EMA recommends using the SIL-IS during LC-MS/MS method validation; but in our study, aciclovir (ARIS) has proven to be an alternative for GCV-d_5_. The total run time of methods presented in this study is no longer than 2 min, which makes them robust (up to 30 analyses per hour). The analytical processes after samples had dried, both for DSS and VAMS in the presented study, took no longer than 40 min to obtain results. Considering the possibility of preparing many samples simultaneously, the short time required for analysis seems relatively attractive. A simple solvent (methanol/water) was used for analyte extraction. Interestingly, the recovery for DSS and VAMS samples increased twofold when sonication was used as an additional step. Samples were purified using the protein precipitation method, and no significant matrix effect was observed. Manual serum sampling is more straightforward in the DSS technique (fixed amount of serum applied to filter paper) than in the VAMS (volumetric) when the fully loaded device tip is not as visible as in blood collecting.

However, DSS and VAMS with dried serum are required upon centrifugation for serum generation, but the generated sample is stable and not required, especially for sample storage. The sample integrity is high, especially in dry ambient conditions with desiccant [24]. Manual serum sampling is simpler for the DSS technique (fixed amount of serum applied to filter paper) than for the VAMS (volumetric) when the fully loaded device tip is less visible than in blood collecting.

The routinely used matrix for GCV determination is plasma, but in some diagnostic centres, serum is more popular for classic biochemical tests. Previously, the Martson et al. method has been calibrated using human serum, similarly to in our study [19]. Additionally, one method for GCV determination in DBS (dried blood spots) was developed, similar to DPS (dried plasma spots) [18,21]. In our study, the LOD in the DSS and VAMS methods was experimentally set to 8 ng/mL, while those in the published DBS and DPS were set to 10 ng/mL and 13 ng/mL, respectively [18,21]. The methods developed in this study yielded attractive stability (7 weeks), whereas the stability evaluated for the DPS method was found to be up to 30 days [21] and only 16 h for the DBS method [18]. Following previously published studies, the ganciclovir exhibited acceptable stability in matrix after five freeze-thaw cycles [18,19,20,21,22,23,24].

In the present study, we decided to use serum instead of plasma because it is routinely collected for TDM of GCV in our laboratory. No significant differences between GCV levels in serum and plasma were observed. Interestingly, we found that the presence of EDTA in plasma (whole blood collected into tubes with potassium salt of EDTA) may improve the stability of GCV, whereas serum collected using a gel separator is not suitable for LC-MS/MS techniques; we have also observed this during analysis.

Our study demonstrates the interchangeability of serum-VAMS and DSS techniques based on satisfactory regression and bias evaluation statistical parameters. Assessment of the DSS technique required accurate manual serum application of serum to the filter paper. The present study has some limitations: namely, the cohort of included patients is relatively homogenous (pediatric patients after renal transplantation only) and the fact that self-sampling process in case of DSS and serum-VAMS samples is not possible outside the laboratory. On the other hand, novel microsampling systems that generate dried plasma in situ after capillary blood collection are greatly needed due to logistic reasons—i.e., sample shipment and storage. TDM tests that are rarely performed may be centralised using DSS/VAMS samples as a means for shipping samples.

## 4. Materials and Methods

### 4.1. Chemicals, Materials and Reagents

The reference standard for ganciclovir (≥99% chemical purity) was acquired from Sigma Aldrich (St. Louis, MO, USA). The stable isotope-labelled internal standard (SIL-IS) and ganciclovir-d_5_ (≥99% isotopic purity) were obtained from Cayman Chemical (Ann Arbor, MI, USA). The structural analogue-related internal standard (ARIS) and aciclovir were purchased from MedChem Tronica (Sollentuna, Sweden). During the experiments, organic solvents characterised by LC-MS purity were used, namely acetonitrile, methanol, and 2-propanol, all produced by Merck (Darmstadt, Germany). Formic acid for LC-MS (>99.99% chemical purity), used as a mobile phase modification, was obtained from Sigma-Aldrich (St. Louis, MO, USA). Zinc sulfate heptahydrate (ZnSO_4_∙7H_2_O, >99.00% chemical purity), used for protein precipitation, was purchased from Sigma-Aldrich (St. Louis, MO, USA). Deionised water for mobile phase preparation was systematically obtained using a Polwater DL90 system (Polwater, Kraków, Poland). LC-MS-grade water LiChrosolv^®^ for stock solution preparation was purchased from Supelco (Bellefonte, PA, USA). Blank human plasma and serum samples from healthy volunteers were obtained from the Regional Centre of Blood Donation and Hemotherapy (Warsaw, Poland). Blood plasma and serum units were separated into smaller amounts (5 mL) and frozen at −20 °C.

Whatman 903 Proteinsaver Cards were obtained from Cytiva (Marlborough, MA, USA). VAMS-Mitra™ samplers (10 µL), clamshells, and cartridges were from Neoteryx, LCC. (Torrance, CA, USA). Chromatographic vials, inserts, and caps were purchased from Alwsci (Zhejiang, China), while other plastic laboratory materials were obtained from GenoPlast Biotech S.A. (Rokocin, Poland).

### 4.2. Stock Solutions

The primary stock solutions for GCV (1000 mg/L), ACV (1000 mg/L), and GCV-d_5_ (100 mg/L) were meticulously prepared using a precise methanol/water (50:50, *v*/*v*) mixture. These solutions were then diluted with the same mix to achieve 100 mg/L and 10 mg/L solutions (for GCV, ACV, GCV, ACV, and GCV-d_5_, respectively). This careful process resulted in fifteen working solutions for calibration and quality control (QC) preparation. The working calibration solutions ranged from 0.05 to 25 mg/L concentrations and were spiked into the plasma. QC samples were prepared using another primary stock solution with concentrations of 0.10, 0.75, 2.50, 7.50, 12.50 and 20.0 mg/L. For calibration, blank plasma obtained from healthy volunteers was used. Stock and working solutions were stored at −20 °C, and calibrators were freshly prepared, ensuring the highest level of accuracy in our research.

### 4.3. LC-MS/MS Analyses

An 8050 triple quadrupole mass spectrometer with a Nexera X2 chromatographic platform (Shimadzu, Kyoto, Japan) was used for the analysis in the present study. The chromatographic system consisted of a gradient binary pump (30AD), degasser device unit (DGU-205AR), autosampler with cooler (SIL-30AC), and oven (CTO-20AC) for column thermostatic maintenance. MS detection was based on a multiple-pair monitoring (MRM) program. Protonated [M + H]^+^ adducts observed in positive ion mode with electrospray ionisation (ESI) were used. The MRM pairs for GCV and internal standards are presented in Table 7.

The detection parameters were optimised experimentally and are listed in Table 8. Nitrogen was used as the drying (DG) and nebulising gas (NG), compressed air as the heating gas (HG), and argon as the gas for collision-induced dissociation (CID).

Chromatographic separation was achieved using an Eclipse Plus C_8_ RRHD column (2.10 × 50 mm, 1.80 µm) from Agilent Technologies, Inc. (Santa Clara, CA, USA). A guarded XDB-C_18_ precolumn (2.1 × 5 mm, 1.80 µm) from the same manufacturer was used. The column was maintained at 30 °C, and a gradient mode mixture of two solutions, (A) water and (B) acetonitrile, both with 0.1% formic acid addition, was used to facilitate the separation process.

(1) 0.01–0.30 min 5% of B phase,
(2) 0.31–1.20 min 75% of B phase,
(3) 1.21–2.00 min 5% of B phase (re-equilibration).

The total run time was 2.00 min with a 0.70 mL/min gradient flow. The autosampler cooler temperature was set at 4 °C for appropriate sample protection. The sample injection speed was equal to 5.00 µL/min, while the needle rinsing speed was 35.00 µL/min in the internal/external mode. The injection volume was set to 0.1 µL for the serum-LC-MS/MS method and to 1 µL for the DSS- and s-VAMS-LC-MS/MS methods.

### 4.4. Sampling Protocol

Serum plasma samples (collected in tubes with a clot activator) were collected from pediatric transplant patients with active CMV infection during a regular preanalytical process for routine TDM of GCV. One hundred microliters of serum were used for all three validated methods presented in the study. Serum (35 µL) was used for the LC-MS/MS method, whereas 50 µL and 10 µL were used for manual DSS and s-VAMS sample preparation, respectively. This study was conducted following the Declaration of Helsinki. The results are part of the standard diagnostic procedure at the Children’s Memorial Health Institute in Warsaw (CMHI). A part of the serum/plasma sample for routine HPLC/UV determination of ganciclovir in Therapeutic Drug Monitoring, Clinical Pharmacokinetics and Toxicology Laboratory (CMHI) was used for the novel methods presented in this study. Additional sample collection was not required for the remainder of the study. Data were blinded and expressed randomly as numbered symbols. The Bioethics Committee of the Children’s Memorial Health Institute in Warsaw was informed about the use of residual routine clinical samples.

### 4.5. Sample Preparation Protocols

#### 4.5.1. Plasma Sample Preparation

To 35 µL of plasma, 3.50 µL of working calibration solution or QC was added. In the case of clinical samples from patients, instead of a calibrator, an equal amount of pure water was added. Next, after sample vortexing, 3.50 µL of the internal standard mixture (final concentration of 12.5 µg/mL for both ACV and GCV-d_5_) was added. Subsequently, 350 µL of precipitation mixture (0.1 M ZnSO_4_:ACN, 1:1, *v*/*v*) was added, and the sample was incubated at −20°C for 20 min. After that sample was centrifuged (1960× *g*, 5 min, 0 °C), 200 µL of clear supernatant was transferred to a clear chromatographic vial, and 0.1 µL was injected into the LC-MS/MS system.

#### 4.5.2. Dried Serum Spot (DSS) Sample Preparation

To 35 µL of plasma, 3.50 µL of working calibration solution or QC was added, vortexed, and carefully pipetted onto the centre of a Whatman filter paper for DSS production. After drying for 2 h under ambient conditions (RT, 23 ± 0.1 °C), each DSS was punched for small spot generation (3.2-mm diameter discs). Each spot was added to 150 µL of water/methanol mixture (1:1, *v*/*v*) and vortexed for 30 min (1000 RPM). In the next step, ultrasound-assisted extraction was used (5 min). After that, 150 µL of precipitation mixture was added, followed by the addition of 3.5 µL of IS solution. Samples were incubated at −20 °C for 20 min and centrifuged (1960× *g*, 5 min, 0 °C) to obtain clear supernatant. The supernatant was transferred to a clear vial, and 1 µL was injected into the LC-MS/MS system.

#### 4.5.3. Dried Serum s-VAMS Sample Preparation

Similar to the DSS sample preparation, the 35 µL serum aliquot was mixed with a calibrator or QC working solution (in the case of the patient sample, an equal amount of water was added CC/QC instead). After vortexing, the 10 µL s-VAMS tips were gently loaded (45° angle, from serum drop on a glass microscope slide). After 2 h of drying, the s-VAMS was extracted using a MeOH/water solution (150 µL) via vortexing and UAE (the same conditions as above). Next, 3.5 µL of IS solution (final concentration 12.5 µg/mL; ACV) was added before 150 µL of precipitation mixture was added. Finally, the sample was incubated at −20 °C for 20 min after vortexing. After centrifugation (1960× *g*, 5 min, 0 °C), 200 µL of clear supernatant was transferred into a glass chromatographic vial, and 1 µL was injected into the LC-MS/MS system.

### 4.6. Method Validation

The European Medicines Agency (EMA) and Food and Drug Administration (FDA) bioanalytical method validation guidelines were assessed during validation [10,11,12,13]. The following parameters were evaluated: specificity, selectivity, lower limit of quantification (LLOQ), limit of detection (LOD), linearity, accuracy, precision, recovery, carry-over effect, matrix effect, and incurred sample bioanalysis (ISR) experiments. Additionally, stability tests under different stress conditions were evaluated (described in Section 4.7). During results evaluation, more rigorous acceptance criteria (EMA vs. FDA) were used [13].

Specificity is the ability to detect and discriminate between analytes and IS accompanied by metabolites, related substances, and other substances in serum. It is widely accepted that any potential interfering compound response should be at most 20% of the analyte and 5% of the IS response in the LLOQ sample [11,12].

Following these guidelines, selectivity was evaluated for blank serum samples (including microsampling methods, without analyte and IS addition) from six individual sources. This parameter is acceptable when no interference is observed at the retention times of the analyte and the IS. Potential interference should not exceed 20% of the analyte and 5% of the IS response in the LLOQ sample [11,12]. The precision of the method, even when two MRMs were used, was high, ensuring accurate measurements.

The lower limit of quantification (LLOQ) is the lowest point of the calibration curve at which an analysis can provide quantitative results. It was estimated during the linearity and calibration curve evaluation.

The limit of detection (LOD) is the lowest concentration determined using the given method. Mathematically, this parameter is calculated using the signal-to-noise (S/N) ratio, where the LOD concentration is estimated as (S/N) equal to 3.0 [11,12,13].

The method’s linearity was assessed in the 0.05–25.0 mg/L calibration range. Each calibration curve was constructed using nine CSs (calibration standards), zero, and a blank sample (without analyte and IS). Based on EMA guidelines, the experiment was repeated six times. It is widely recognised that the mean R^2^ should be higher than 0.95 [8]. Each calibration curve was evaluated using 1/× weighting.

Accuracy and precision parameters were evaluated using QC samples (LLOQ—0.10 mg/L, MQC_1_—0.75 mg/L; MQC_2_, 7.50 mg/L; and HQC—20.0 mg/L). Accuracy was calculated as the percentage ratio of the determined value to the reference (nominal) value, and precision was expressed as the percentage coefficient of variation (CV%). Both parameters were evaluated within a run (intra-run, n = 10) and between runs (inter-day, n = 10). EMA acceptance criteria were established as ±15% of the nominal value for accuracy (±20% for LLOQ), whereas the CV% for precision evaluation should be lower than 15% (20% for LLOQ) [11,12,13].

The EMA criteria do not mention the acceptable range for recovery. In present study, this parameter was evaluated at two different QC levels (n = 6) by spiking GCV into aliquots of plasma (or during microsampling treatment procedures) before and after extraction [11].

The presence of a potential carry-over effect was evaluated by injection of a blank sample promptly after ULOQ (25 mg/L) analysis. The EMA acceptance criterion was fulfilled when the response for the analyte and IS was not greater than 20% and 5% of the reaction at LLOQ, respectively [11,12,13].

The matrix effect (ME) was evaluated in post-extraction experiments. ME, AR, and PE were expressed as percentage ratios and were calculated following the methodology of Taylor et al. and Matuszewski et al. [14,15]. Experiments were based on six repetitions of sample analysis at two QC levels. The analyte and IS responses were assessed in ACN-enriched samples and in the presence of the matrix.

Adhering to EMA guidelines, ISR experiments were performed considering the presence of metabolites, concomitant drugs, and non-homogeneity in the analysed sample [11]. The experiment involved repeating the assay for 10% of the samples included in the study and calculating the parameter description as the ratio of differences between the results to the mean value of the measurements. The accepted criterion is for at least 67% of the repetitions to meet the acceptance criterion of 20% of the mean, ensuring regulatory compliance in our ISR experiments [11].

### 4.7. Stability Testing

Stability evaluation is a crucial element of method validation, especially according to the analyte stability in the matrix collected from the patient. Stability was considered acceptable when the measured concentration was within ±15% of the nominal value. For this purpose, the following stability experiments were performed at two QCs levels in triplicate [11,12,13]:

- Autosampler stability (analysed at 24 h, 72 h, and 120 h after preparation),
- Working solution stability (analysed after one, three, and six months of storage at −20 °C),
- Freeze-thaw stability in the matrix (minimum of three freeze-thaw cycles, performed after a minimum of 12-h storage in the freezer),
- Bench-top (short-term) stability in the matrix (stored at ambient temperature, before and after extraction for 2 h and 4 h compared to a standard set of samples),
- Long-term stability in matrix during storage at room temperature (analysed after one, two, four, five, and seven weeks counted from the initial assay).

### 4.8. HPLC/UV Method for GCV Determination

As a routine approach, the previously validated and optimised HPLC/UV method is used for GCV determination in the Therapeutic Drug Monitoring, Clinical Pharmacokinetics, and Toxicology Laboratory at the Children’s Memorial Health Institute in Warsaw, Poland. For analysis, Varian Inc. (Palo Alto, CA, USA), a ProStar M230 gradient pump combined with a ProStar M325 UV-VIS detector (190–900 nm measurement range) was used. As a stationary phase, Ascentis C_18_ (250 × 4.6 mm, 5 µm) chromatographic column with complementary guard pre-column Ascentis C_8_ (20 × 4.0 mm, 5 µm), both from Supelco (Bellefonte, PA, USA), was used as the stationary phase. The analysis was conducted under isocratic conditions using a mobile phase consisting of water, acetonitrile, 0.5 M potassium dihydrogen phosphate solution, and phosphoric acid (460:39:0.1:0.9, *v*/*v*/*v*/*v*) with 1.60 mL/min as a flow rate. A wavelength of 254 nm was used for analyte and internal standard (aciclovir) detection. Clinical samples were prepared using the following protocol: 100 µL of serum was mixed with 50 µL of aciclovir (20 mg/L), and acetonitrile (1.0 mL) was added. The extraction process was supported by sonication. The supernatant obtained during centrifugation (1370× *g*) was evaporated to dryness under nitrogen steam at 70 °C. Fifty microlitres of the residue dissolved in the mobile phase was injected into the column. The limit of detection of the described method was set to 0.25 mg/L, and factor (F; equal 0.94) was used for GCV concentration calculations using the formula: GCV concentration [mg/L] = (GCV peak area/IS peak area) × F.

### 4.9. Statistical Analysis

For chromatogram analysis, MRMs optimisation, calibration curve weighting, signal-to-noise evaluation, and method calibration, dedicated LabSolutions software (version 5.98; Shimadzu, Tokyo, Japan) were used. Passing-Bablok regression, Bland-Altman bias evaluation, and correlation methods were performed using MedCalc software (version 22.026, MedCalc, Ostend, Belgium), Statistica (version 13.3, StatSoft Inc., Kraków, Poland), and MS Excel (version 13.65, Microsoft Corporation, Redmond, WA, USA).

## 5. Conclusions

A novel, rapid, highly sensitive LC-MS/MS method is proposed for GCV determination in serum, DSS, and VAMS samples. The wide assay range ensures the possibility of GCV determination at trough and peak levels or in pharmacokinetic studies. The methods validated in the study are applicable to TDM of GCV in populations where CMV infection is relatively frequent and dangerous: namely, neonates and SOT recipients. Furthermore, aciclovir perfectly compensated matrix effect. ACV turned out to be more stable over the long-term than SIL-IS. On the other hand, there is a risk of ACV presence in patient samples (when administrated concomitantly with (V)GCV). Therefore, it may be an alternative IS for SIL-IS, reducing the analysis cost.

In summary, the fast, simple, and sensitive LC-MS/MS method may be routinely used in daily clinical practice for TDM of GCV. DSS and VAMS devices ensure high analyte stability; thus, the presented technique simplifies the logistic process of sample transfer. Importantly, we demonstrated the comparability of the three validated methods to the routinely used HPLC/UV, providing reassurance about the reliability of our method. Due to the wide calibration range, the presented methodologies may be used in pharmacokinetics studies, which are still needed in some populations, including infants and children. Additionally, the validated methods provide a foundation for future GCV determination in VAMS collected capillary blood (including self-sampling) method optimization and validation.

To our knowledge, this is the first application of VAMS-Mitra™ to determine GCV in dried serum.

## Figures and Tables

**Table 1:** Results of intra-run and between-run accuracy and precision examination [n = 10].

Parameter	LLOQ—0.10 mg/L				MQC_1_—0.75 mg/L				MQC_2_—7.50 mg/L				HQC—20.00 mg/L			
	Serum IS: GCV-d_5_	Serum IS: ACV	DSS IS: ACV	s-VAMS IS: ACV	Serum IS: GCV-d_5_	Serum IS: ACV	DSS IS: ACV	s-VAMS IS: ACV	Serum IS: GCV-d_5_	Serum IS: ACV	DSS IS: ACV	s-VAMS IS: ACV	Serum IS: GCV-d_5_	Serum IS: ACV	DSS IS: ACV	s-VAMS IS: ACV
**Within-run (intra-day) accuracy and precision [n = 10]**																
C_GCV_ [mg/L]	0.11 ± 0.02	0.10 ± 0.01	0.12 ± 0.02	0.52 ± 0.04	0.77 ± 0.05	0.76 ± 0.03	0.72 ± 0.04	0.76 ± 0.03	7.13 ± 0.46	7.45 ± 0.14	7.38 ± 0.19	7.50 ± 0.24	20.20 ± 0.82	19.99 ± 0.10	20.17 ± 0.49	20.12 ± 0.27
Accuracy [%]	107.76	104.41	103.61	103.96	101.50	105.23	96.66	103.12	100.59	97.83	98.46	99.86	99.80	99.15	99.74	100.18
Precision [%]	16.26	10.77	17.87	14.33	5.92	3.99	5.58	4.18	5.49	2.41	2.60	3.24	4.08	0.51	2.42	1.33
**Between-run (inter-day) accuracy and precision [n = 10]**																
C_GCV_ [mg/L]	0.11 ± 0.01	0.10 ± 0.01	0.12 ± 0.02	0.11 ± 0.02	0.75 ± 0.04	0.77 ± 0.05	0.77 ± 0.05	0.77 ± 0.05	7.67 ± 0.24	7.63 ± 0.18	7.49 ± 0.14	7.46 ± 0.26	20.67 ± 0.41	20.13 ± 0.24	20.22 ± 0.19	20.18 ± 0.20
Accuracy [%]	102.29	104.41	103.61	105.59	99.19	101.50	101.50	101.50	100.20	100.24	100.01	99.48	100.55	99.82	100.00	100.46
Precision [%]	11.80	10.77	17.87	13.64	5.33	5.92	5.92	5.92	3.08	2.36	1.92	3.51	1.98	1.21	0.92	0.99

Data are expressed as mean ± standard deviation (SD; with min/max range); LLOQ—lower limit of quantification; MQC—medium quality control, HQC—higher quality control, IS—internal standard, GCV-d_5_—deuterated ganciclovir, ACV—aciclovir.

**Table 2:** Autosampler stability of samples of serum, DSS, and s-VAMS samples using the LC-MS/MS method.

GCV Concentration [mg/L]	Calculated Concentration [mg/L] and Stability at 4 °C [%]			
	Initial Day (T = 0)	After 24 h	After 72 h	After 120 h
**IS: GCV-d_5_ [n = 6]—LC-MS/MS method in serum**				
LLOQ (0.10)	0.12 ± 0.04; 100.00	0.11 ± 0.05; 93.67	0.12 ± 0.06; 95.96	0.10 ± 0.08; 91.33
MQC_1_ (0.75)	0.76 ± 0.08; 100.00	0.74 ± 0.05; 97.86	0.74 ± 0.07; 97.96	0.72 ± 0.12; 93.38
MQC_2_ (7.50)	7.54 ± 0.23; 100.00	7.55 ± 0.20; 100.09	7.49 ± 0.32; 97.87	7.48 ± 0.29; 96.94
HQC (20.00)	20.09 ± 1.01; 100.00	20.13 ± 1.21; 101.99	20.06 ± 1.19; 98.90	19.37 ± 1.65; 94.86
**IS: ACV [n = 6]—LC-MS/MS method in serum**				
LLOQ (0.10)	0.11 ± 0.03; 100.00	0.09 ± 0.04; 89.81	0.09 ± 0.03; 90.11	0.09 ± 0.05; 89.87
MQC_1_ (0.75)	0.76 ± 0.11; 100.00	0.72 ± 0.13; 94.76	0.73 ± 0.14; 94.23	0.74 ± 0.19; 95.02
MQC_2_ (7.50)	7.52 ± 0.19; 100.00	7.54 ± 0.23; 101.57	7.57 ± 0.25; 103.21	7.34 ± 0.33; 96.23
HQC (20.00)	20.23 ± 1.34; 100.00	20.59 ± 1.45; 101.26	19.98 ± 1.61; 98.87	18.59 ± 2.16; 93.43
**IS: ACV [n = 6]—LC-MS/MS method in DSS**				
LLOQ (0.10)	0.14 ± 0.06; 100.00	0.12 ± 0.07; 89.71	0.10 ± 0.03; 81.43	0.09 ± 0.01; 74.69
MQC_1_ (0.75)	0.77 ± 0.12; 100.00	0.75 ± 0.18; 98.77	0.70 ± 0.17; 91.87	0.67 ± 0.18; 87.29
MQC_2_ (7.50)	7.58 ± 0.36; 100.00	7.54 ± 0.31; 98.25	7.42 ± 0.38; 96.88	7.09 ± 0.41; 93.62
HQC (20.00)	20.34 ± 1.98; 100.00	20.19 ± 1.69; 97.95	19.22 ± 1.93; 94.19	18.93 ± 2.31; 90.38
**IS: ACV [n = 6]—LC-MS/MS method in s-VAMS**				
LLOQ (0.10)	0.11 ± 0.02; 100.00	0.09 ± 0.03; 89.99	0.10 ± 0.03; 90.79	0.09 ± 0.02; 87.37
MQC_1_ (0.75)	0.74 ± 0.13; 100.00	0.73 ± 0.14; 98.34	0.76 ± 0.20; 101.97	0.69 ± 0.18; 97.49
MQC_2_ (7.50)	7.58 ± 0.29; 100.00	7.59 ± 0.33; 98.76	7.44 ± 0.66; 98.15	7.26 ± 0.73; 96.07
HQC (20.00)	20.16 ± 0.99; 100.00	20.49 ± 1.71; 102.48	20.06 ± 1.28; 99.05	19.09 ± 1.44; 96.99

Data are expressed as the mean concentration ± SD; LC-MS/MS—liquid chromatography-tandem mass spectrometry, GCV—ganciclovir, GCV-d_5_—deuterated ganciclovir, ACV—aciclovir, DSS—dried serum spots, s-VAMS—dried serum in volumetric-absorptive microsampling.

**Table 3:** Bench-top experiment for LC-MS/MS methods using serum, DSS, and s-VAMS samples [n = 6].

GCV Concentration [mg/L]	Calculated Concentration [mg/L] and Stability at RT [%]											
	Serum (IS: GCV-d_5_) [n = 6]			Serum (IS: ACV) [n = 6]			DSS (IS: ACV) [n = 6]			s-VAMS(IS: ACV) [n = 6]		
	0 h	−2 h	+2 h	0 h	−2 h	+2 h	0 h	−2 h	+2 h	0 h	−2 h	+2 h
MQC_1_ (0.75)	0.74 ± 0.08 100.00%	0.71 ± 0.07 95.59%	0.73 ± 0.09 98.33%	0.72 ± 0.09 100.00%	0.73 ± 0.06 101.23%	0.76 ± 0.08 102.37%	0.74 ± 0.02 100.00%	0.74 ± 0.04 99.13%	0.71 ± 0.06 96.91%	0.74 ± 0.08 100.00%	0.76 ± 0.09 101.70%	0.75 ± 0.03 99.64%
MQC_2_ (7.50)	7.79 ± 0.46 100.00%	7.58 ± 0.48 97.10%	7.31 ± 0.37 95.93%	7.65 ± 0.54 100.00%	7.55 ± 0.41 96.20%	7.71 ± 0.39 99.81%	7.61 ± 0.21 100.00%	7.58 ± 0.48 97.28%	7.42 ± 0.29 93.46%	7.46 ± 0.18 100.00%	7.51 ± 0.23 100.98%	7.33 ± 0.31 97.11%

Data are expressed as mean ± SD (with min/max range); RT, room temperature (~23°C), LC-MS/MS—liquid chromatography-tandem mass spectrometry, GCV—ganciclovir, GCV-d_5_—deuterated ganciclovir, ACV—aciclovir, DSS—dried serum spots, s-VAMS—dried serum in volumetric-absorptive microsampling.

**Table 4:** Results of matrix effect, process efficiency, and absolute recovery post-extraction experiments [n = 6].

Parameter	LLOQ—0.10 mg/L			MQC_1_—0.75 mg/L			MQC_2_—7.50 mg/L			HQC—20.00 mg/L		
	GCV	IS	F	GCV	IS	F	GCV	IS	F	GCV	IS	F
**serum-LC-MS/MS [n = 6] IS: GCV-d_5_**												
ME [%]	−12.25 ± 2.21	−16.24 ± 2.73	−0.92 ± 0.67	−17.89 ± 4.24	−14.31 ± 3.06	−1.29 ± 0.24	−13.21 ± 3.89	−12.88 ± 2.98	1.09 ± 0.19	−10.43 ± 5.89	−12.36 ± 3.41	−0.89 ± 0.19
PE [%]	68.93 ± 2.87	68.10 ± 3.46	99.32 ± 5.21	70.09 ± 7.21	72.22 ± 5.01	100.09 ± 7.21	64.23 ± 4.87	66.93 ± 5.87	97.34 ± 2.07	77.31 ± 4.68	73.03 ± 2.99	100.33 ± 5.07
AR [%]	74.26 ± 1.56	78.55 ± 2.24	98.79 ± 7.75	69.57 ± 2.34	78.23 ± 3.14	96.23 ± 4.01	69.89 ± 3.76	73.58 ± 4.94	98.31 ± 2.17	70.63 ± 5.24	63.10 ± 3.39	98.76 ± 6.40
**serum-LC-MS/MS [n = 6] IS:ACV**												
ME [%]	−13.98 ± 3.54	−15.27 ± 6.34	0.89 ± 0.56	−12.55 ± 4.31	−9.99 ± 2.14	1.04 ± 0.78	−13.39 ± 6.98	−16.15 ± 11.23	−0.76 ± 0.34	−9.74 ± 3.26	−13.13 ± 6.12	1.12 ± 0.87
PE [%]	70.42 ± 4.47	67.67 ± 5.41	102.62 ± 10.51	65.34 ± 7.23	66.56 ± 9.95	93.15 ± 7.99	67.22 ± 8.14	71.72 ± 6.69	94.56 ± 3.67	78.22 ± 5.67	73.75 ± 4.24	100.91 ± 11.99
AR [%]	66.20 ± 6.25	59.33 ± 7.22	103.33 ± 4.68	68.11 ± 4.43	66.15 ± 10.79	98.95 ± 8.48	65.22 ± 6.36	67.33 ± 5.03	98.26 ± 4.16	68.78 ± 7.21	71.34 ± 5.34	99.01 ± 12.44
**DSS-LC-MS/MS [n = 6] IS:ACV**												
ME [%]	−14.83 ± 6.12	−12.37 ± 4.81	0.79 ± 0.31	−14.39 ± 6.41	−12.22 ± 4.89	1.02 ± 0.91	−16.21 ± 5.90	−14.05 ± 6.16	0.95 ± 0.64	−10.11 ± 6.32	−9.99 ± 4.78	1.45 ± 1.05
PE [%]	66.57 ± 4.78	63.39 ± 4.57	101.21 ± 8.76	64.75 ± 5.09	67.98 ± 10.11	96.01 ± 4.01	63.33 ± 8.39	67.21 ± 5.78	96.06 ± 8.91	68.98 ± 7.94	70.77 ± 7.35	100.01 ± 6.23
AR [%]	60.01 ± 7.45	58.11 ± 9.54	98.99 ± 10.12	72.33 ± 8.43	70.14 ± 12.72	97.05 ± 5.48	64.67 ± 7.01	67.00 ± 6.23	98.01 ± 7.86	67.57 ± 6.78	70.98 ± 5.34	97.97 ± 3.22
**s-VAMS-LC-MS/MS [n = 6] IS:ACV**												
ME [%]	−11.32 ± 7.00	−10.11 ± 7.81	−1.09 ± 0.82	−10.09 ± 6.21	−8.96 ± 5.21	1.00 ± 0.57	−11.66 ± 4.72	−11.09 ± 5.76	0.89 ± 0.42	−10.01 ± 2.87	−11.87 ± 6.79	1.22 ± 0.72
PE [%]	71.31 ± 8.12	72.07 ± 14.22	102.62 ± 10.23	70.77 ± 5.98	72.42 ± 7.01	97.01 ± 5.89	68.01 ± 7.52	69.87 ± 8.01	98.94 ± 6.12	70.54 ± 7.12	71.07 ± 5.55	97.56 ± 8.56
AR [%]	70.33 ± 6.66	75.12 ± 5.99	99.99 ± 12.01	76.53 ± 7.01	78.05 ± 6.43	98.52 ± 6.04	70.78 ± 5.29	70.13 ± 6.86	98.01 ± 3.31	73.24 ± 5.87	75.12 ± 6.31	96.831 ± 5.92

Data are presented as arithmetic mean ± standard deviation, F—factor (GCV peak area/IS peak area ratio); ME: matrix effect; PE: process efficiency; AR: absolute recovery; LC-MS/MS—liquid chromatography-tandem mass spectrometry, GCV—ganciclovir, GCV-d_5_—deuterated ganciclovir, ACV—aciclovir, DSS—dried serum spots, s-VAMS—dried serum in volumetric-absorptive microsampling.

**Table 5:** Long-term stability of DSS and s-VAMS stored at RT [n = 6].

GCV Concentration [mg/L]	Calculated Concentration [mg/L] and Stability at RT [%]											
	DSS (IS: ACV) [n = 6]						s-VAMS (IS: ACV) [n = 6]					
	T = 0	7 Days	14 Days	28 Days	35 Days	49 Days	T = 0	7 Days	14 Days	28 Days	35 Days	49 Days
MQC_1_ (0.75)	0.74 ± 0.05 100.00%	0.75 ± 0.08 98.95%	0.70 ± 0.11 95.16%	0.68 ± 0.09 91.44%	0.67 ± 0.06 89.76%	0.66 ± 0.09 87.79%	0.75 ± 0.04 100.00%	0.75 ± 0.06 99.33%	0.73 ± 0.05 96.57%	0.70 ± 0.13 93.08%	0.68 ± 0.19 89.76%	0.67 ± 0.23 85.86%
MQC_2_ (7.50)	7.56 ± 0.28 100.00%	7.54 ± 0.18 97.89%	7.31 ± 0.22 95.93%	7.16 ± 0.54 93.61%	6.89 ± 0.41 92.01%	6.69 ± 0.72 89.41%	7.52 ± 0.19 100.00%	7.49 ± 0.25 100.29%	7.43 ± 0.31 97.17%	7.37 ± 0.36 96.01%	7.24 ± 0.31 90.27%	7.03 ± 0.57 87.28%

Data are presented as arithmetic mean ± standard deviation, with mean percentage stability compared to initial concentration (T = 0). ACV—aciclovir, DSS—dried serum spots, s-VAMS—dried serum in volumetric-absorptive microsampling, RT—room temperature.

**Table 6:** Summary of statistics in cross-validation procedure between paired GCV concentration results determined using chromatographic methods.

Statistical Tool	Compared Methods					
	Serum-LC-MS/MS (GCV-d_5_ as IS) versus Serum-LC-MS/MS (ACV as IS)	Serum-LC-MS/MS (ACV as IS) versus DSS-LC-MS/MS (ACV as IS)	Serum-LC-MS/MS (ASC as IS) versus s-VAMS-LC-MS/MS (ACV as IS)	DSS-LC-MS/MS (ACV as IS) versus s-VAMS-LC-MS/MS (ACV as IS)	Serum-LC-MS/MS (GCV-d_5_ as IS) versus Serum-HPLC-UV (ACV as IS)	Serum-LC-MS/MS (ACV as IS) versus Serum-HPLC-UV (ACV as IS)
Passing-Bablok regression formula	y = 0.996x + 0.003	y = 1.048x − 0.013	y = 1.072x − 0.011	y = 1.019x + 0.019	y = 0.997x + 0.021	y = 1.009x − 0.075
Intercept (A)	0.003 (−0.032 to 0.053)	−0.013 (−0.073 to 0.066)	−0.011 (−0.058 to 0.029)	0.019 (−0.029 to 0.058)	0.021 (−0.010 to 0.083)	−0.075 (−0.183 to −0.008)
Slope (B)	0.996 (0.935 to 1.075)	1.048 (0.930 to 1.155)	1.072 (0.984 to 1.125)	1.019 (0.981 to 1.067)	0.997 (0.917 to 1.048)	1.009 (0.920 to 1.112)
Bland-Altman bias [%]	4.53% (−0.71 to 9.77)	3.56% (−4.56 to 11.72)	−3.61% (−8.38 to 1.16)	−9.06% (−15.29 to −2.82)	5.87% (−2.65 to 14.39)	12.34% (2.20 to 22.48)
% of paired samples fulfilled EMA criteria (*bias* < 20%)	67.25%	72.50%	82.50%	86.25%	63.25%	61.25%
Pearson’s correlation coefficient (R^2^)	0.96 (0.93 to 0.97)	0.94 (0.90 to 0.96)	0.94 (0.91 to 0.96)	0.97 (0.95 to 0.98)	0.98 (0.97 to 0.99)	0.93 (0.89 to 0.95)
Spearman rank correlation coefficient (SRCC)	0.99 (0.98 to 0.99)	0.97 (0.96 to 0.98)	0.98 (0.98 to 0.99)	0.99 (0.98 to 1.00)	0.98 (0.96 to 0.99)	0.96 (0.93 to 0.97)

GCV—ganciclovir, ACV—aciclovir, LC-MS/MS—liquid chromatography tandem mass spectrometry, HPLC-UV—high-performance liquid chromatography with ultraviolet detection, DSS—dried serum spots, VAMS—volumetric-absorptive microsampling.

**Table 7:** Multiple pair monitoring (MRM) transition for analytes: The first pair was used for quantification, and the second for qualitative determination in each case.

Analyte	Monitored Adduct	MRM Pairs (*m*/*z*)	CE [eV]	Dwell Time [ms]
GCV	[GCV + H]^+^	256.05 → 152.00 256.05 → 135.10	−20 −12	17 17
GCV-d_5_	[(GCV-d_5_) + H]^+^	261.15 → 152.05 261.15 → 135.10	−13 −12	17 17
ACV	[ACV + H]^+^	226.10 → 152.00 226.10 → 135.00	−15 −27	17 17

GCV—ganciclovir, GCV-d_5_—deuterated ganciclovir, ACV—aciclovir, CE—collision energy.

**Table 8:** Mass spectrometry detection parameters used for analysis.

Parameter	Value
electrospray voltage [kV]	1.00
nebulizing gas [L/min]	2.50
drying gas [L/min]	5.00
heating gas [L/min]	7.00
interface temperature [°C]	200
desolvation temperature [°C]	355
desolvation line temperature [°C]	200
heat block temperature [°C]	300
CID pressure [kPa]	270

CID—collision-induced dissociation process.

## Data Availability

The authors will make the raw data supporting this article’s conclusions available upon request.
